# Chemical shift assignments of the α-actinin C-terminal EF-hand domain bound to a cytosolic C0 domain of GluN1 (residues 841–865) from the NMDA receptor

**DOI:** 10.1007/s12104-024-10194-2

**Published:** 2024-08-29

**Authors:** Aritra Bej, Johannes W. Hell, James B. Ames

**Affiliations:** 1 Departments of Chemistry, University of California, Davis, CA 95616 USA; 2 Departments of Pharmacology, University of California, Davis, CA 95616 USA

**Keywords:** a-actinin, Calcium, GluN1, NMDA receptor, C0 domain, NMR

## Abstract

N-methyl-D-aspartate receptors (NMDARs) consist of glycine-binding GluN1 and glutamate-binding GluN2 subunits that form tetrameric ion channels. NMDARs in the brain are important for controlling neuronal excitability to promote synaptic plasticity. The cytoskeletal protein, α-actinin-1 (100 kDa, called ACTN1) binds to the cytosolic C0 domain of GluN1 (residues 841–865) that may play a role in the Ca^2+^-dependent desensitization of NMDAR channels. Mutations that disrupt NMDAR channel function are linked to Alzheimer’s disease, depression, stroke, epilepsy, and schizophrenia. NMR chemical shift assignments are reported here for the C-terminal EF-hand domain of ACTN1 (residues 824–892, called ACTN_EF34) and ACTN_EF34 bound to the GluN1 C0 domain (BMRB numbers 52385 and 52386, respectively).

## Biological context

Synaptic transmission and its plasticity in the brain is governed by Ca^2+^-dependent regulation of NMDA receptors that serve as ligand-gated Na^+^/Ca^2+^ channels (Traynelis et al. 2010). Different NMDA receptor subtypes are comprised of tetrameric combinations of glycine binding GluN1 and glutamate binding GluN2A-D subunits (A-D) subunits (Benveniste and Mayer 1991; Clements and Westbrook 1991) that assemble as a 2:2 complex, (GluN1)_2_:(GluN2)_2_. The ligand-gated opening of NMDAR channels leads to neuronal Ca^2+^ influx (Wadel et al. 2007), which promotes various Ca^2+^-dependent processes (Kunz et al. 2013; Puri 2020). Prolonged elevation of the intracellular Ca^2+^ level is cytotoxic (Peng et al. 1998), and NMDAR channels are negatively regulated by a process known as Ca^2+^-dependent channel inactivation (CDI) (Zhang et al. 1998) mediated by ACTN1 (Krupp et al. 1999; Rycroft and Gibb 2004; Shaw and Koleske 2021) and calmodulin (Iacobucci and Popescu 2017, 2019, 2020) The Ca^2+^-induced desensitization of NMDAR channels requires binding of both ACTN1 and calmodulin to the cytosolic C0 domain in GluN1 (Iacobucci and Popescu 2017, 2019, 2020; Zhang and Majerus 1998). The C-terminal EF-hand domain of ACTN1 (residues 824–892, called ACTN_EF34) does not bind to Ca^2+^ in the physiological range (Backman 2015; Turner et al. 2020). Instead, the Ca^2+^-free EF-hand domain competes with CaM for binding to the IQ-motif in the CaV1.2 L-type Ca^2+^ channel (Turner et al. 2020). We hypothesize a similar competitive binding of ACTN1 to C0 in GluN1 may promote conformational changes in NMDARs that control channel desensitization (Iacobucci and Popescu 2020; Krupp et al. 1999; Wang et al. 2008).

Recent cryo-EM structures of NMDA receptors (Chou et al. 2020; Jalali-Yazdi et al. 2018; Karakas and Furukawa 2014; Lee et al. 2014; Regan et al. 2018) reveal structural interactions between the extracellular amino-terminal and ligand-binding domains, and their coupling to the transmembrane channel domain. However, the C-terminal cytosolic domain of GluN1 (involved in channel desensitization) is not structurally defined in the available structures. The cytosolic region of GluN1 contains a predicted helical C0 domain (residues 841–865) that binds to CaM (Ehlers et al. 1996) and ACTN1 (Merrill et al. 2007; Rycroft and Gibb 2004; Wyszynski et al. 1997). We recently reported NMR assignments of CaM bound to the GluN1 C0 domain (Bej and Ames 2023). We report here NMR chemical shift assignments of ACTN_EF34 bound to the GluN1 C0 domain. These assignments provide a basis for elucidating the structure of ACTN1 bound to GluN1, which may provide insights into channel desensitization.

## Methods and experiments

*Preparation of ACTN_EF34 bound to GluN1 C0.* The third and fourth EF-hands of human α-actinin-1 (residues 824–892, called ACTN_EF34) were subcloned into pET15b expression vector (Novagen) and overexpressed in E. coli strain BL21(DE3) that produced recombinant N-terminal 6xHis-tagged (MGSSHHHHHSSGLVPRGSHM) ACTN_EF34 protein. Uniformly ^13^C,^15^N-labeled ACTN_EF34 samples were obtained as described previously (Turner et al. 2020) by growing cells in M9 minimal media supplemented with ^15^NH_4_Cl (1 g/L) and ^13^C-labled D-glucose (3 g/L) (Cambridge Isotopes Laboratories). The soluble fraction of the cell lysate was loaded onto a HisTrap HP column pre-equilibrated with wash buffer (20 mM Tris (pH 8.0), 500 mM NaCl, 10 mM imidazole, 1 mM β-mercaptoethanol) and eluted at 300 mM imidazole. The eluted fraction containing ACTN_EF34 was loaded onto a HiPrep Q Sepharose anion exchange column pre-equilibrated with 50 mM Tris (pH 8.0), 25 mM KCl, 1 mM EGTA, 1 mM DTT and eluted using a linear KCl gradient (0 to 625 mM). The purity and identity of the eluted protein fractions were confirmed by sodium dodecyl sulfate-polyacrylamide gel electrophoresis (SDS-PAGE). A peptide fragment of the GluN1 C0 domain (residues 841–865) was purchased from GenScript and samples were prepared as described previously (Bej and Ames 2023). A 1.5-fold excess of peptide was added to ACTN_EF34, incubated at room temperature for 30 min, and concentrated to 0.5 mM in a final volume of 0.5 ml.

**Fig. 1:**
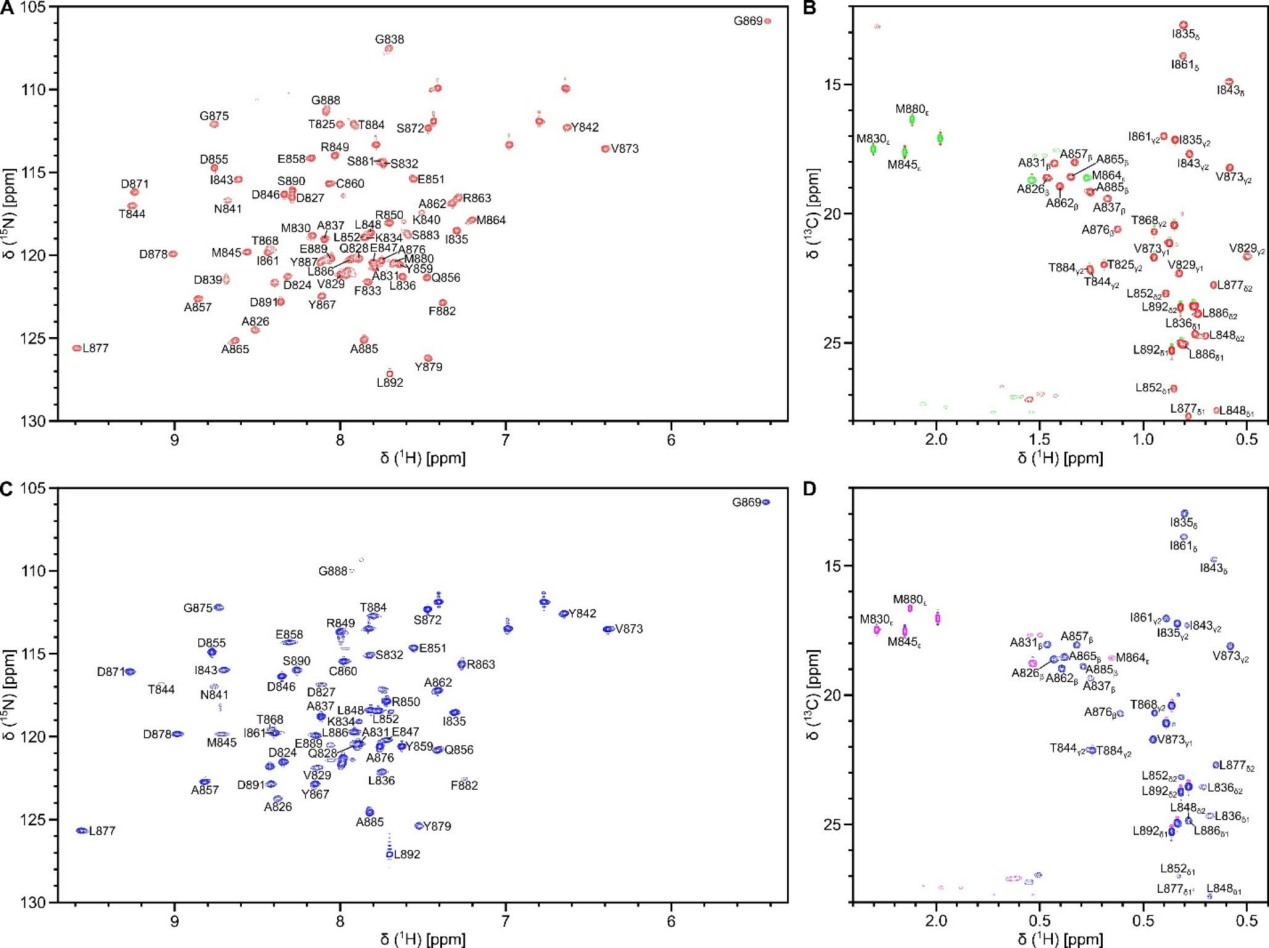
Backbone and side chain resonance assignments of free ACTN_EF34 and ACTN_EF34 bound to GluN1 C0 peptide. ^15^N-^1^H HSQC spectra of free ACTN_EF34 (**A**) and ACTN_EF34/C0 (**C**) illustrate backbone assignments indicated by labeled peaks. Constant-time ^13^C-^1^H HSQC spectra of free ACTN_EF34 (**B**) and ACTN_EF34/C0 (**D**) illustrate side chain methyl assignments. All spectra recorded at 800 MHz ^1^H frequency on samples that contained 0.5 mM ^13^C,^15^N-labeled ACTN_EF34 (bound to unlabeled C0 peptide) in 20 mM Tris-d_11_ (pH 7.5), 1 mM EDTA-d_12_, and 1 mM DTT-d_10_ at 302 K

*NMR spectroscopy.* NMR samples of Ca^2+^-free forms of free ACTN_EF34 (or ACTN_EF34/C0) were dissolved in 20 mM Tris-d_11_ (pH 7.5), 1 mM EDTA-d_12_, and 1 mM DTT-d_10_ containing 8% or 100% (v/v) D_2_O and packed into precision NMR tubes (Wilmad). NMR experiments on ACTN_EF34 and ACTN_EF34/C0 were performed at 302 K on a Bruker Avance III 800 MHz spectrometer equipped with a four-channel interface and triple resonance cryogenic (TCI) probe. The ^15^N-^1^H HSQC spectra (Fig. 1A and C) were recorded with 256 × 2048 complex points for ^15^N(F1) and ^1^H(F2). Triple resonance NMR experiments (HNCACB, HN(CO)CACB, HNCO, HBHA(CO)NH, and HBHANH) were performed and analyzed to assign the backbone resonances. CC(CO)NH, H(CCO)NH, HCCH-TOCSY, HBCBCGCDHD, HBCBCGCDCEHE, and ^13^C-edited NOESY-HSQC were analyzed to assign the side chain resonances. All NMR data were processed using NMRPipe (Delaglio et al. 1995) and assignment was performed using Sparky (Lee et al. 2015).

## Extent of assignments and data deposition

The ^15^N-^1^H HSQC spectra of Ca^2+^-free forms of ^13^C, ^15^N-labeled ACTN_EF34 (Fig. 1A) and ^13^C, ^15^N-labeled ACTN_EF34 bound to unlabeled C0 (called ACTN_EF34/C0 in Fig. 1C) illustrate backbone resonance assignments for the 64 non-proline residues (excluding the N-terminal 6xHis-tag and thrombin cleavage site: MGSSHHHHHSSGLVPRGSHM). The highly resolved ^15^N-^1^H HSQC peaks with uniform intensities suggest that free ACTN_EF34 and ACTN_EF34/C0 both adopt a stable and folded structure. All 64 non-proline resonances were assigned for free ACTN_EF34, and 52 out of 64 non-proline amide resonances were assigned for ACTN_EF34/C0, indicated by the labeled peaks in Fig. 1A and C. The unassigned amide resonances (for residues T825, M830, F833, G838, D839, K840, M864, A865, M880, S881, S883, and Y887) have very weak NMR intensities, perhaps caused by exchange broadening due to interactions with the C0 peptide. The amide resonances assigned to G869 and V873 exhibited noteworthy upfield shifts in both free ACTN_EF34 (Fig. 1A) and ACTN_EF34/C0 (Fig. 1C), because these residues are flanked by nearby aromatic rings of Y842 and Y867. Side chain aliphatic resonance assignments of free ACTN_EF34 (Fig. 1B) and ACTN_EF34/C0 (Fig. 1D) are illustrated by the labeled peaks in the constant-time ^13^C-^1^H HSQC spectra (Fig. 1B and D). The chemical shift assignments (^1^H, ^13^C, and ^15^N) for free ACTN_EF34 and ACTN_EF34/C0 were deposited in the BioMagResBank (http://www.bmrb.wisc.edu) under accession number 52385 and 52386, respectively.

**Fig. 2:**
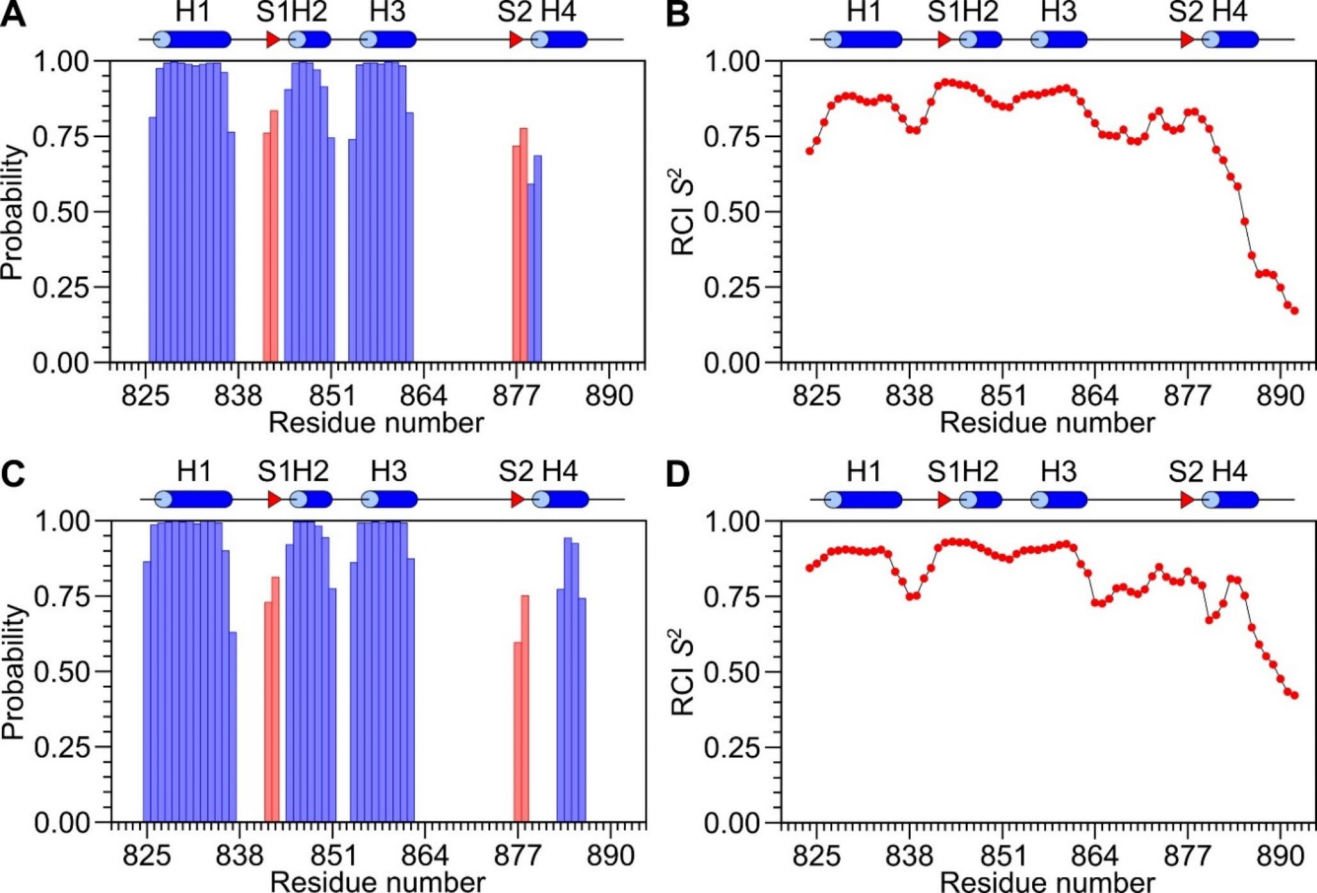
Secondary structure and random-coil index order parameters (RCI S^2^) analysis of free ACTN_EF34 and ACTN_EF34/C0 using TALOS + server (Shen et al. 2009). Probability of secondary structural elements of free ACTN_EF34 (**A**) and ACTN_EF34/C0 (**C**). Blue cylinders represent α-helices and red arrows indicate β-strands derived from the NMR structure of ACTN_EF34 bound to the IQ-motif from the L-type Ca^2+^ channel (PDB ID: 6C0A). The predicted RCI S^2^ of free ACTN_EF34 (**B**) and ACTN_EF34/C0 (**D**)

Based on backbone chemical shifts (^1^HN, ^15^N, ^13^Cα, ^13^Cβ, ^13^CO), secondary structural elements and random-coil index order parameters (RCI S^2^) were predicted using the TALOS + server (Shen et al. 2009) (Fig. 2). The secondary structure of free ACTN_EF34 and ACTN_EF34/C0 are very similar to previous NMR structures of ACTN1 (Drmota Prebil et al. 2016; Turner et al. 2020). The secondary structure of both free ACTN_EF34 and ACTN_EF34/C0 has four α-helices (blue cylinders in Fig. 2A and C): H1 (residues 825–837), H2 (residues 845–851), H3 (residues 854–864), and H4 (residues 879–888) as well as two short β-strands (red triangles in Fig. 2A and C): S1 (residues 842–843) and S2 (residues 877–878) located in the loop region of the two EF-hands as seen in previous structures of ACTN1 (Drmota Prebil et al. 2016; Turner et al. 2020). The C-terminal helix (H4) is 4 residues longer in ACTN_EF34/C0 compared to that of free ACTN_EF34. The shorter H4 helix in free ACTN_EF34 might be explained by the dynamical nature of the C-terminal region, which has RCI S^2^ values of less than 0.6 (Fig. 2B) compared to higher RCI S^2^ values for ACTN_EF34/C0 (Fig. 2D). These results suggest that C0 binding to ACTN_EF34 stabilizes the H4 helix.

**Fig. 3:**
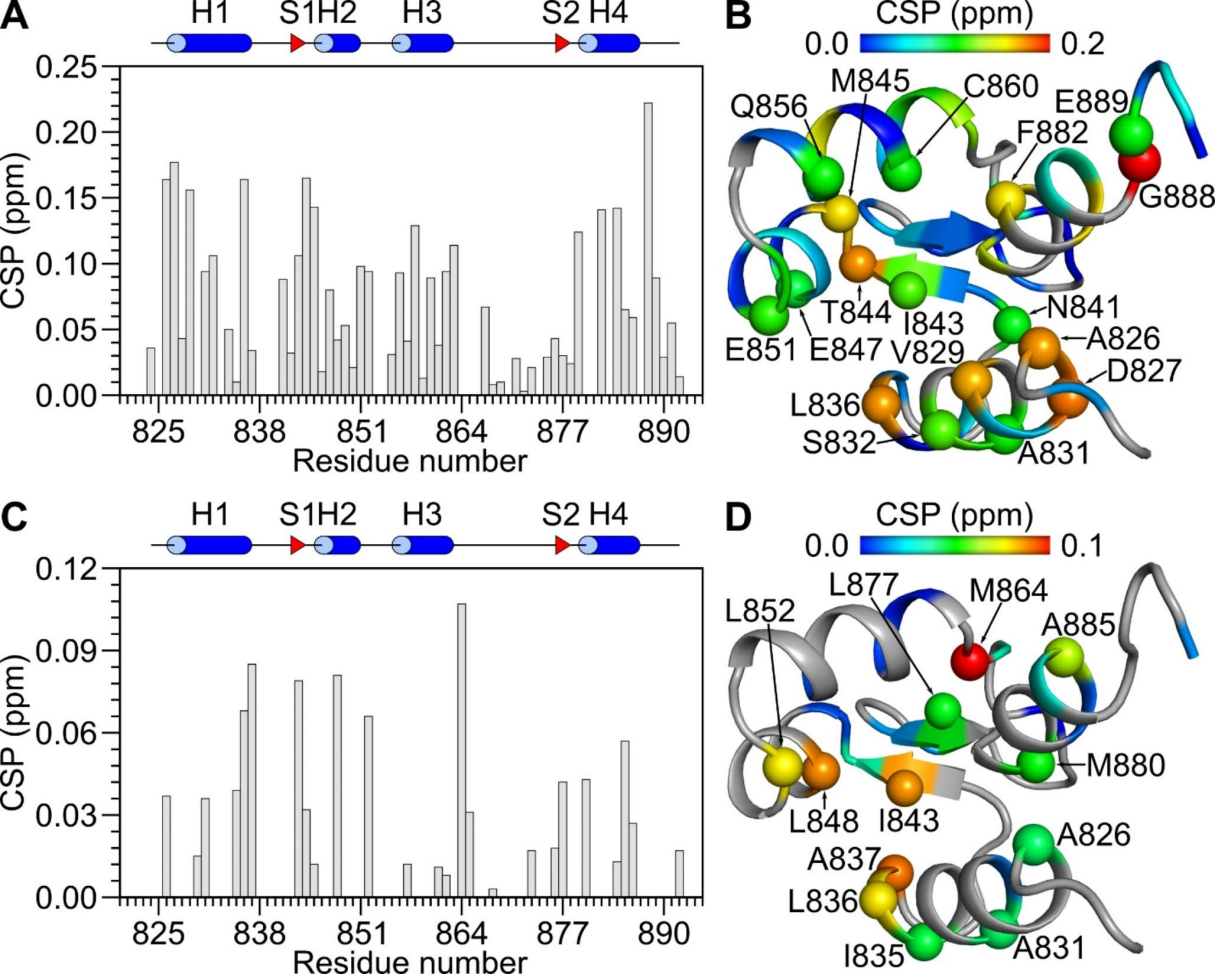
Chemical shift perturbation (CSP) for free ACTN_EF34 versus ACTN_EF34/C0. CSP values of backbone amide resonances (**A**) were calculated as: $\:\text{C}\text{S}\text{P}=\:\sqrt{{\left({\varDelta\:\text{H}}^{\text{N}}\right)}^{2}+{(0.14\times\:\varDelta\:\text{N})}^{2}}$, where ΔH^N^ and ΔN are the observed difference in the amide ^1^H and ^15^N chemical shifts, respectively between free ACTN_EF34 and ACTN_EF34/C0. CSP values of side chain methyl resonances (**C**) were calculated as: $\:\text{C}\text{S}\text{P}=\:\sqrt{{(\varDelta\:H)}^{2}+{(0.3\times\:\varDelta\:\text{C})}^{2}}$ (Williamson 2013), where ΔH and ΔC are the observed difference in the methyl ^1^H and ^13^C chemical shifts, respectively between free ACTN_EF34 and ACTN_EF34/C0. CSP values of backbone amide resonances (**B**) and side chain methyl resonances (**D**) are mapped onto the ACTN1 structure (PDB ID: 6C0A, chain A (Turner et al. 2020). Residues with largest CSP values are shown as spheres and labeled accordingly. Residues, without CSP values including proline, amino acids without methyl group, or unassigned resonances, are colored gray

A comparison of chemical shifts between free ACTN_EF34 and ACTN_EF34/C0 suggests residues in ACTN_EF34 that may interact with the bound C0 peptide (Fig. 3A and C). The largest chemical shift perturbations (CSPs) were observed for exposed residues (A826, V829, A831, L836, I843, L848, L852, M864, L877, M880, F882, and A885) in both EF-hands of ACTN_EF34 that cluster to form a potential GluN1 binding site (see spheres in Fig. 3B and D). Indeed, these same exposed residues of ACTN_EF34 (A826, V829, L836, L852, and F882) interact with the CaV1.2 IQ peptide in the previous NMR structure of ACTN1 bound to CaV1.2 IQ (Turner et al. 2020). We propose the exposed hydrophobic crevice in ACTN_EF34 (Fig. 3B and D) may interact with a helical C0 peptide like what is seen in the ACTN1/IQ NMR structure.

## Data Availability

The NMR chemical shift assignments have been deposited to the Biologic Magnetic Resonance Data Bank under the accession codes 52385 and 52386.
